# Steric nested AS‐PCR detects SNP in the W1282X‐CFTR allele with endpoint thermal cycler

**DOI:** 10.14814/phy2.71165

**Published:** 2026-11-08

**Authors:** John A. Torok, Derrick L. Brown, Michael R. Grassl, Samyuktha Vijayakumar, Kierin P. Patel, Ayaan Jehangir, Jay Katti, Daniel F. Barnas, Logan P. Dooley, Anahita Dhar, Chase A. MacDougall, Ryan J. Ciacci, Siersha R. Torres, Nicole Katrib, Douglas Luckie

**Affiliations:** ^1^ Lyman Briggs College East Lansing Michigan USA; ^2^ Department of Physiology Michigan State University East Lansing Michigan USA

**Keywords:** accessible, cystic fibrosis, diagnostic, low‐cost, mutation

## Abstract

To develop a cost‐effective approach, allele‐specific and nested polymerase chain reaction (PCR) techniques were combined with a steric hindrance destabilization strategy to improve discrimination of DNA samples positive for W1282X‐CFTR using an affordable endpoint PCR thermal cycler. Using Yaku's steric strategy, forward primers contained a deliberate mismatch near the mutation site. We hypothesized that these allele‐specific primers could selectively amplify DNA containing the W1282X mutation while reducing amplification of non‐matching wild‐type sequences. Use of low‐cost endpoint PCR methods supported diagnostic accessibility across more healthcare facilities. Primary PCR utilized nested forward and reverse primers to generate a 591 bp template for secondary PCR. Secondary PCR employed designed wild‐type and mutant forward primers alongside a universal reverse primer targeting the nested product. Positive results were found for mutant and wild‐type DNA targets. The strategy was further validated against a second SNP mutation target, G542X‐CFTR. These findings support those in the literature and demonstrate the feasibility of nested allele‐specific endpoint PCR as a rapid, cost‐effective approach for the most difficult‐to‐detect single‐base mutations. This method may serve as a more affordable diagnostic approach for detecting SNPs in any gene target, potentially reducing reliance on specialized diagnostic platforms.

## 1 INTRODUCTION

Endpoint polymerase chain reaction (PCR) is a traditional method now used for more affordable identification of a targeted gene DNA sequence (Cruz‐Rangel et al., 2022; Gustafson et al., 1993). During the first stage, purified double‐stranded DNA is heated to denature the hydrogen bonds between the nucleotides, separating the sample into single strands (Garibyan & Avashia, 2013). The temperature is then lowered to a temperature conducive for sequence‐specific forward and reverse primers to bind complementary target DNA segments. In the final stage, the temperature is raised so the polymerase adds nucleotides to the end of each primer (Erlich, 1989). These three stages run for 25–30 cycles, allowing for desired amplification (Lee et al., 2012). Agarose gel electrophoresis is typically used to visualize DNA products (Lee et al., 2012). While numerous more complex applications of the PCR strategy are common in professional research, the traditional, simpler end‐point approach may be much more accessible for rural healthcare in the US and elsewhere due to less expensive equipment and supplies required.

Allele‐specific (AS) PCR can be used to detect the presence of known DNA sequences of genes from a variety of sources. In this study, it was applied to determine the genotype of human DNA samples for a genetic disease. Cystic Fibrosis (CF) is an autosomal recessive disorder caused by mutations in the cystic fibrosis transmembrane conductance regulator (CFTR) gene (Fanen et al., 2014), affecting approximately 80,000 individuals worldwide. The CFTR gene is located on chromosome 7 and encodes the anion channels responsible for cAMP‐regulated chloride transport across the apical membranes of epithelial cells (Riordan et al., 1989). A healthy CFTR protein works as a chloride ion channel (Rowntree & Harris, 2003). Impaired CFTR function disrupts ion transport and fluid homeostasis in epithelial tissues, resulting in thick mucus, chronic pulmonary infections, and progressive lung damage (Castellani & Assael, 2016). More than 2100 mutant variants of the CFTR gene have been identified to date (Petrova et al., 2021). Among these, several mutations are commonly associated with CF, including F508del, G542X, N1303K, G551D, W1282X, 1717‐1G → A, R553X, 621 + 1G → T, and R1162X. The W1282X mutation is the sixth most common CFTR mutation worldwide, accounting for approximately 1.2% of mutant CFTR alleles. It occurs at a particularly high frequency within the Ashkenazi Jewish population (Michicich et al., 2025).

W1282X is designated as a severe Class I CFTR mutation, in which a nonsense mutation introduces a premature stop codon. The resulting premature termination of translation leads to production of a truncated CFTR protein, leading to reduced levels of functional CFTR protein and impaired chloride channel efficiency (Rowe et al., 2007). The mutation arises from a G → A substitution at codon 1282 in exon 23, converting the tryptophan (W) codon TGG into the stop (X) codon TGA (Suzuki et al., 2022) The W1282X mutation incapacitates CFTR function by reduced steady‐state levels of CFTR mRNA and, consequently, levels of CFTR protein, which occurs through a mechanism of nonsense‐mediated decay (NMD) (Wang et al., 2016). W1282X‐CFTR polypeptides produced despite NMD will most likely have reduced channel activity. This is because the mutation truncates the protein in the second cytoplasmic nucleotide binding domain (NBD2), critical for efficient CFTR function (Wang et al., 2016).

## 2 METHODS

### 2.1 Materials

Low‐cost Taq polymerase enzyme was obtained from Syd Labs *MB042‐EUT*. The 100 bp Gold Bio MW ladder *D001‐500* and agarose powder *A‐201‐100* were obtained from Gold Biotechnology. A 100 bp DNA Ladder *15628019* and SYBR‐Safe™ DNA gel stain *S33102* were purchased from Invitrogen. The lithium borate electrophoresis buffer *LB20‐1* was obtained from Faster Better Media. Oligonucleotide DNA primers were purchased from IDT. Cell samples were obtained from You et al. (2024).

### 2.2 PCR primers

#### 2.2.1 PCR Reagent Control

As a positive control for PCR reagent functionality, the Rz gene of the Lambda bacteriophage genome was amplified using two Rz gene primers. 1Rz1F was a Rz gene forward primer: 5′‐GATGTATGAGCAGAGTCA CCGCGAT‐3′ and 1Rz1R was a Rz gene reverse primer: 5′‐GAGGGTGAAATAATCCC GTTCAG‐3′ (Table 1). As designed, this produced a PCR product of 395 bases.

**TABLE 1:** Primers used in PCR amplification of Lambda control, published primer control, and Yaku sterically designed allele‐specific primers.

Primer	Primer abbreviation	Primer sequence (5′–3′)
“Designed” Wild‐Type Forward Yaku Primer	WTF‐Yaku1	TCAATAACTTTGCAACAGCGG
“Designed” Mutant Type Forward Yaku Primer	MF‐Yaku1	TCAATAACTTTGCAACAGCGA
“Designed” Δ Mutant Type Forward Yaku Primer	ΔMF‐Yaku1	CAGAGAAAGACAATATAGTTCATT
Published Reverse Primer & Universal Reverse	PRprim1‐URP	CTATGAGAAAACTGCACTGGA
Δ Published Reverse Primer & Universal Reverse	ΔPRprim1‐URP	GCACAGATTCTGAGTAACCATAAT
Published Forward Primer & Nested Forward Primer	PFprim1‐NestF	GGTCAGGATTGAAAGTGTGCA
“Designed” Nested Reverse Primer	DNestR1	AACCTGGCGATTGCTAACTTG
Lambda Rz Gene Forward Primer	Rz1F	GATGTATGAGCAGAGTCACCGCGAT
Lambda Rz Gene Reverse Primer	Rz1R	GAGGGTGAAATAATCCCGTTCAG

#### 2.2.2 DNA Genome Control

As a positive control for CFTR gene DNA sequence integrity, additional published primer sequences were used. PFprim1‐NestF was a published forward primer: 5′‐GGTCAGGATTGAAAGTGTGCA‐3′ and PRprim1‐URP was a published reverse primer: 5′‐CTATGAGAAAACTGCACTGGA‐3′. As designed, this produced a PCR product of 471 bases (Table 1). DNA primer sequences were obtained from Feriotto et al. (2001).

#### 2.2.3 Nested‐PCR

A nested PCR strategy was employed using the forward primer sequence, PFprim1‐NestF: 5′‐GGTCAGGATTGAAAGTGTGCA‐3′ and reverse primer sequence, DNestR1: 5′‐AACCTGGCGATTGCTAACTTG‐3′. As designed, these primers amplified a 651‐base template region (Table 1 and Figure 1). DNA primer sequences were obtained from Feriotto et al. (2001).

**FIGURE 1:**
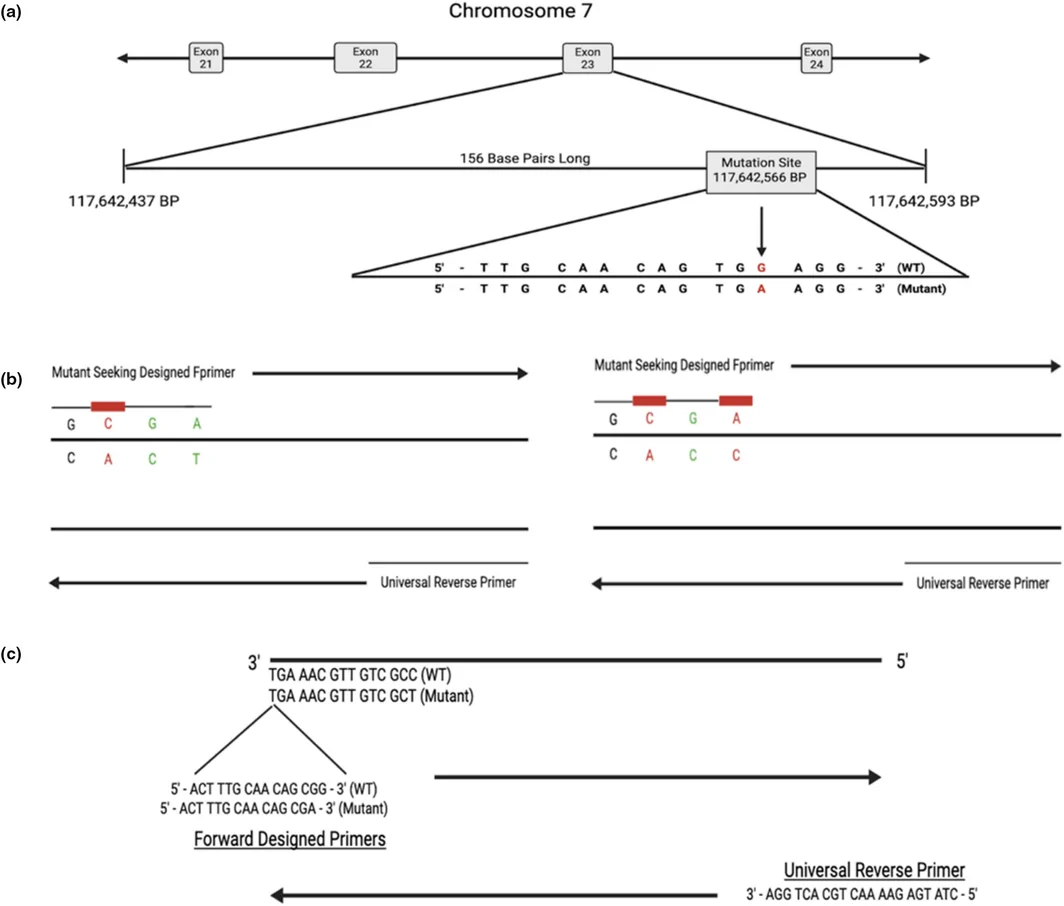
Genomic localization, allele‐specific primer discrimination, and expected PCR outcomes for the W1282X CFTR mutation. (a) Schematic of the CFTR gene on chromosome 7q31.2 highlighting the W1282X nonsense mutation within exon 23. (b) Mechanistic illustration of the steric allele‐specific primer design strategy based at the final three‐base (“X–Y–Z” nucleotide positions) architecture of the primer. (left) When mutant‐seeking Fprimer binds at its intended target, the W1282X mutant allele, an intentional mismatch at the “X” position is tolerated when correct pairing occurs at the 3′ terminal “Z” nucleotide, permitting selective extension and amplification of W1282X alleles. (right) In contrast, inappropriate amplification of wild‐type DNA by mutant‐seeking Fprimer is suppressed because mismatches at both the “X” and “Z” positions destabilize the entire 3′ end and prevent polymerase extension. (c) Expected allele‐specific PCR amplification outcomes for W1282X genotypes. Due to base‐pair specificity, the wild‐type primer is expected to amplify wild‐type and heterozygous templates, whereas the mutant primer is expected to amplify mutant and heterozygous templates. Successful amplification yields an approximately 190 bp product, with single‐band amplification observed for homozygous templates and dual‐band amplification expected for heterozygous DNA.

### 2.3 Allele‐specific primer design

Allele‐specific primers were designed according to Hidenobu Yaku's intentional single base pair 3′ substitution strategy when targeting SNPs with endpoint PCR (Yaku et al., 2008). A mechanistic diagram is provided to illustrate how non‐specific amplification is suppressed (Figure 1).

#### 2.3.1 AP‐PCR

Allele‐specific primers were designed following the Yaku steric method of introducing intentional base pair mismatches near the 3′ end. A wild‐type forward primer sequence WTF‐Yaku‐1: 5′‐TACATAACTTTGCAACAGGGG‐3′, and a mutant forward primer sequence MF‐Yaku‐1: 5′‐TCAATAACTTTGCAACAGCGA‐3′ were designed to work with a universal reverse primer: 5′‐CATTGAGAAAACTGCACTGGA‐3′ and produce a 190‐base PCR product (Table 1 and Figure 1).

### 2.4 Cell culture

S9 is a stable cell line that expresses the W1282X‐CFTR gene. S9 cell lines were grown in Dulbecco's Modified Eagle's Medium (DMEM) supplemented with 10% fetal bovine serum (FBS), 2 mM glutamine, 100 units/mL penicillin, and 10 μg/mL streptomycin.

### 2.5 DNA purification

Purified genomic DNA samples were obtained with the use of Chelex‐100 resin. A 10% stock solution of Chelex‐100 resin was composed of 0.10 grams of Chelex resin in 1.0 mL of DNase‐free water. A 1.5 mL sample of approximately 1 × 10^6^ cells was centrifuged at 14000 rpm for 2 min until a pellet was formed. Supernatant was removed, and a solution of 500 μL of 10% Chelex was added and vortexed until the pellet was resuspended. The sample was incubated in a 56°C water bath for 10 min and was vortexed for 5 s every 5 min. After 10 min, the sample was placed into a 100°C bath for 5 min. The solution was centrifuged one last time at 14000 rpm for 5 min to form a Chelex pellet. The supernatant containing purified DNA was separated from the Chelex pellet and stored at −20°C until use. A 260/280 analysis was performed to measure nucleic acid concentration and purity of DNA samples.

### 2.6 Polymerase chain reaction

To conduct PCR, a 50 μl cocktail was created using 35.8 μl of nuclease‐free H_2_O, 5.0μl of 10× PCR buffer (200 mM Tris–HCl (pH 8.4) and 500 mM KCl), 1.5 μl of 25 mM MgCl_2_ and 1.0μl of each; 10 mM dNTP's, 100 μM forward primer, 100 μM reverse primer, 1ug/μl DNA template, and 5 U/μl taq polymerase. A T100 Bio‐Rad thermal cycler was used. An initial denaturing temperature of 95°C was used for 3 min. Thirty cycles were used with: (1) a denaturing temperature of 95°C for 30 s, (2) an annealing stage for 45 s, (3) a polymerization temperature of 72°C for 1 min.

### 2.7 Gel electrophoresis

A 1% agarose gel was used to perform gel electrophoresis. The materials included 0.5 g of agarose, 2.5 mL of LB 20× (200 mM lithium borate) buffer, 47.5 mL of dH_2_O, and 5 μL of 10,000× SYBR‐Safe™ dye. The mixture of dH_2_O, 20× LB buffer, and agarose powder was warmed in the microwave until dissolved. After the mixture was cooled slightly, SYBR‐Safe™ dye was added, and the gel cast. 6X loading dye containing 30% glycerol, 0.25% (0.025 g/10 mL) bromophenol blue, and 0.25% (0.025 g/10 mL) xylene cyanol was used during loading of samples. A 1X LB running buffer solution was used during electrophoresis at 150 V for 30 min. The gel was analyzed and photographed under UV light.

### 2.8 Data analysis

Gel electrophoresis was analyzed by comparing the migration of bands in the molecular weight ladder to those of PCR products using a semi‐logarithmic plot. A trendline was added and an *R* ^2^ value was obtained. The trendline equation was used to calculate PCR product base‐pair lengths.

## 3 RESULTS

An allele‐specific PCR assay for the detection of the W1282X CFTR mutation was designed for evaluation alongside control PCR primers (Table 1). Allele‐specific primers were designed using the Yaku steric mismatch strategy (Figure 1).

### 3.1 Evaluation of PCR reagents

A Rz gene positive control was used regularly to assess the functionality of reagents and was designed to produce a single band of 395 base pairs (Figure 2). In the sample gel highlighted, a single band was observed at the migration distance of 2.767 cm. To calculate the base pair (bp) length of the PCR product, the molecular weight ladder was used as a reference. From the measurements taken, a semi‐logarithmic graph was generated, and the PCR product was calculated to be approximately 396 bp (Figure 2b). The close correlation between the observed migration distance and the predicted fragment length supported the functionality of the endpoint PCR reagents and protocols.

**FIGURE 2:**
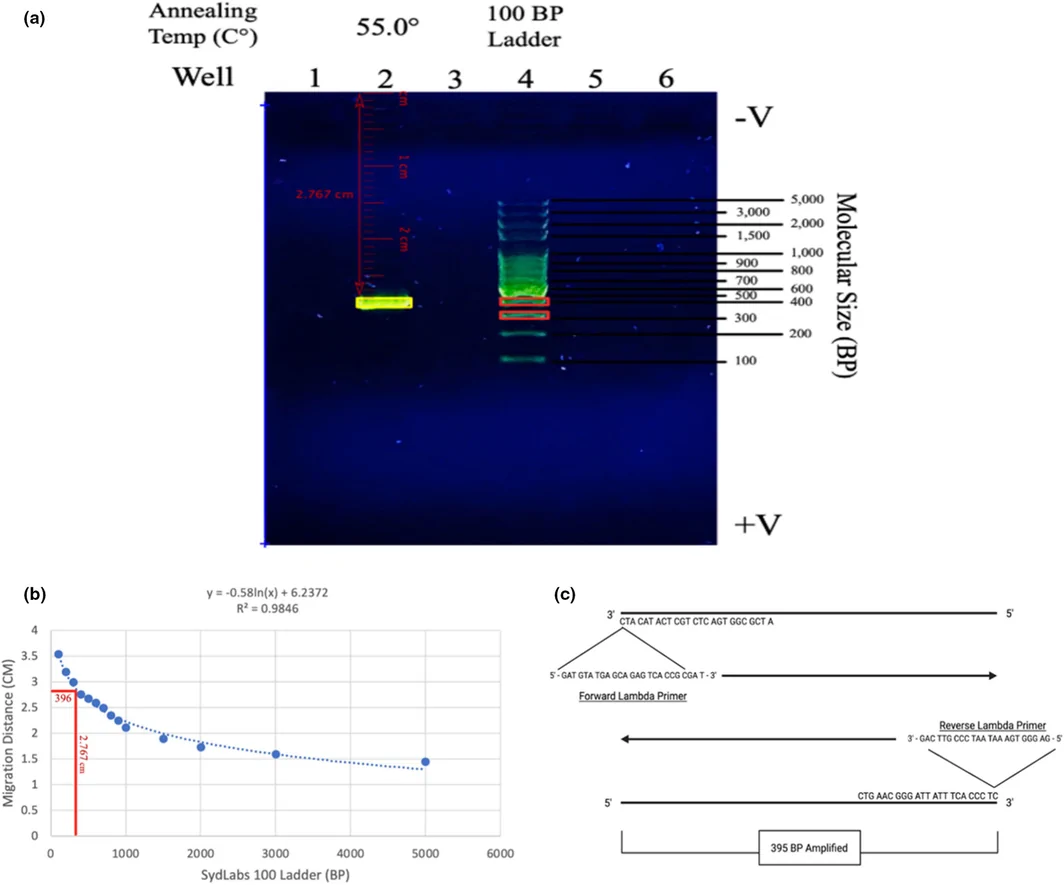
Control PCR of lambda viral DNA using published Rz primers. (a) Gel electrophoresis showing amplification of the Rz gene from lambda viral DNA using published primers. A single band near ~395 bp represents the expected amplicon, supporting that PCR components and thermocycling conditions were functional. A 100 bp molecular weight ladder was included as a reference. (b) Semi‐logarithmic analysis of band migration distance from agarose gel electrophoresis. The measured migration distance of 2.767 cm corresponded to a calculated fragment length of ~396 bp using the ladder‐derived standard curve, confirming amplification of the expected control amplicon. (c) Schematic showing the annealing positions of the Rz forward and reverse primers on the lambda viral genome. The forward primer binds starting at 45,961 bp, while the reverse primer binds ending at 46,356 bp on the opposite strand, generating a 395 bp product.

### 3.2 Assessment of purified DNA

Genomic DNA extracted from human buccal epithelial cells was evaluated by UV–Vis spectrophotometry. DNA yield and purity varied between trials; however, trial 3 produced the highest concentration (207 ng/μL) and the most favorable A260/280 ratio (1.59), consistent with acceptable protein‐free genomic DNA. This sample was selected as the wild‐type CFTR template for subsequent control and allele‐specific PCR assays.

### 3.3 Verification of CFTR locus integrity

PCR amplification of the exon 23 region using primers “Fprim‐1” and “URprim‐1” was used to verify the presence of wild‐type CFTR genomic DNA in purified samples. In gel electrophoresis, amplification of genomic DNA produced a single band migrating near the expected position for a 471 bp amplicon in gel electrophoresis (Figure 3). Semi‐logarithmic analysis yielded a line of best fit equations to calculate the base pair length of the amplified DNA (Figure 3b), where y represents the migration distance (2.403 cm) and x represents the molecular size. A calculated fragment size of 472 bp (Figure 3b) supported the predicted amplicon was obtained. A schematic of primer annealing in introns 22 and 23 is provided (Figure 3c).

**FIGURE 3:**
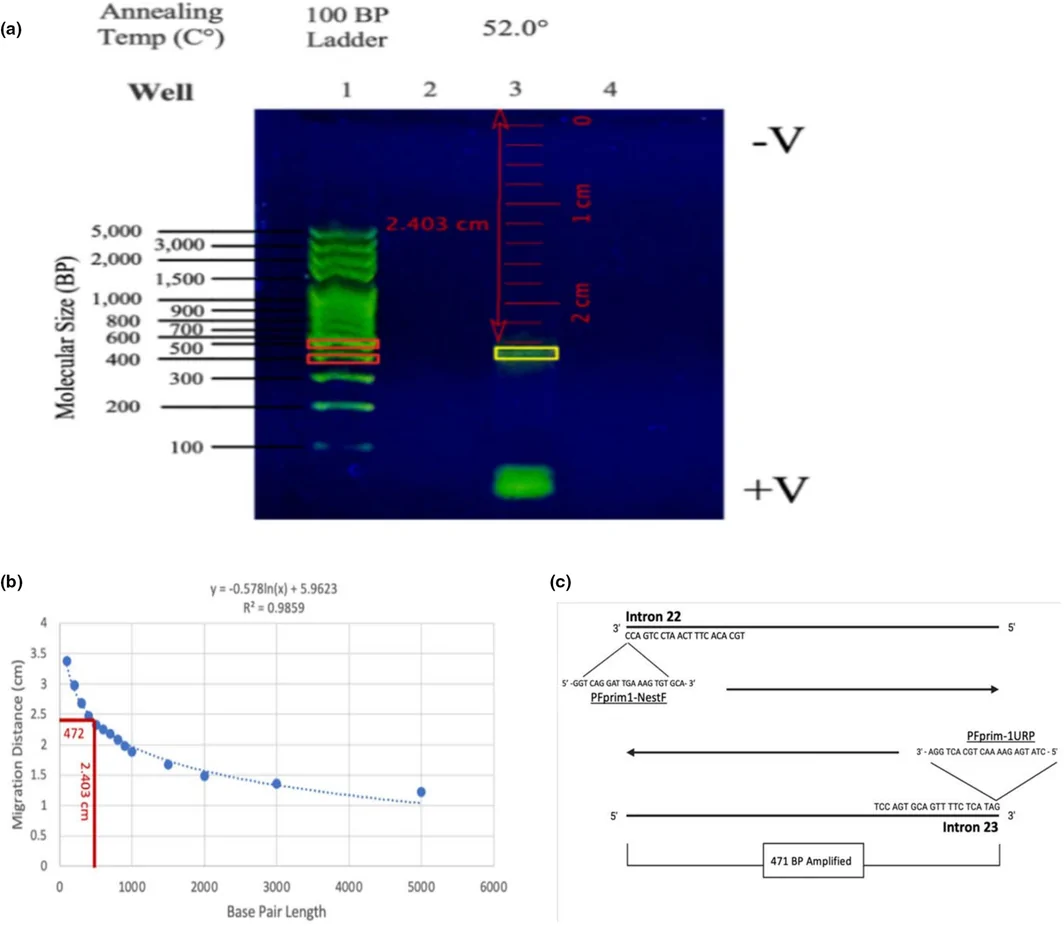
Wild‐type CFTR amplification using published control primers. (a) Agarose gel demonstrating amplification of a ~471 bp wild‐type CFTR amplicon from buccal genomic DNA using published primers flanking exon 23. (b) Semi‐logarithmic sizing analysis calculated a product length of 472 bp, in agreement with the predicted amplicon size. (c) Schematic of Fprim‐1 and URprim‐1 primer binding sites within introns 22 and 23, respectively, generating a control amplicon that spans exon 23 and the W1282X locus.

### 3.4 Allele‐specific amplification

Experimental PCR analysis demonstrated strong allele specificity consistent with predicted outcomes. When wild‐type–seeking primers were paired with wild‐type CFTR template, a single ~190 bp amplicon was observed, with no detectable amplification from W1282X mutant DNA (Figure 4). In contrast, mutant‐seeking primers produced a single ~190 bp band exclusively from W1282X mutant template, with complete suppression of amplification from wild‐type DNA (Figure 5). These mutually exclusive amplification patterns support allele discrimination by the designed primer sets. The molecular basis for this selectivity is illustrated in Figure 1, which illustrates how intentional mismatches at the three 3′ terminal (referred to as X, Y, Z) primer positions inhibit 3′‐end extension on non‐target alleles while permitting extension on correctly paired templates. To confirm product identity, migration distances of allele‐specific amplicons were analyzed using semi‐logarithmic regression relative to molecular weight ladder standards. Both wild‐type–specific and mutant‐specific PCR products resolved to calculate fragment lengths of approximately 190 bp, consistent with predicted amplicon size (Figure 4). This analysis validates the identity of products observed on agarose gels (Figure 5) and supports steric mismatch‐based discrimination. The primer–template interactions responsible for these validated outcomes are summarized schematically.

**FIGURE 4:**
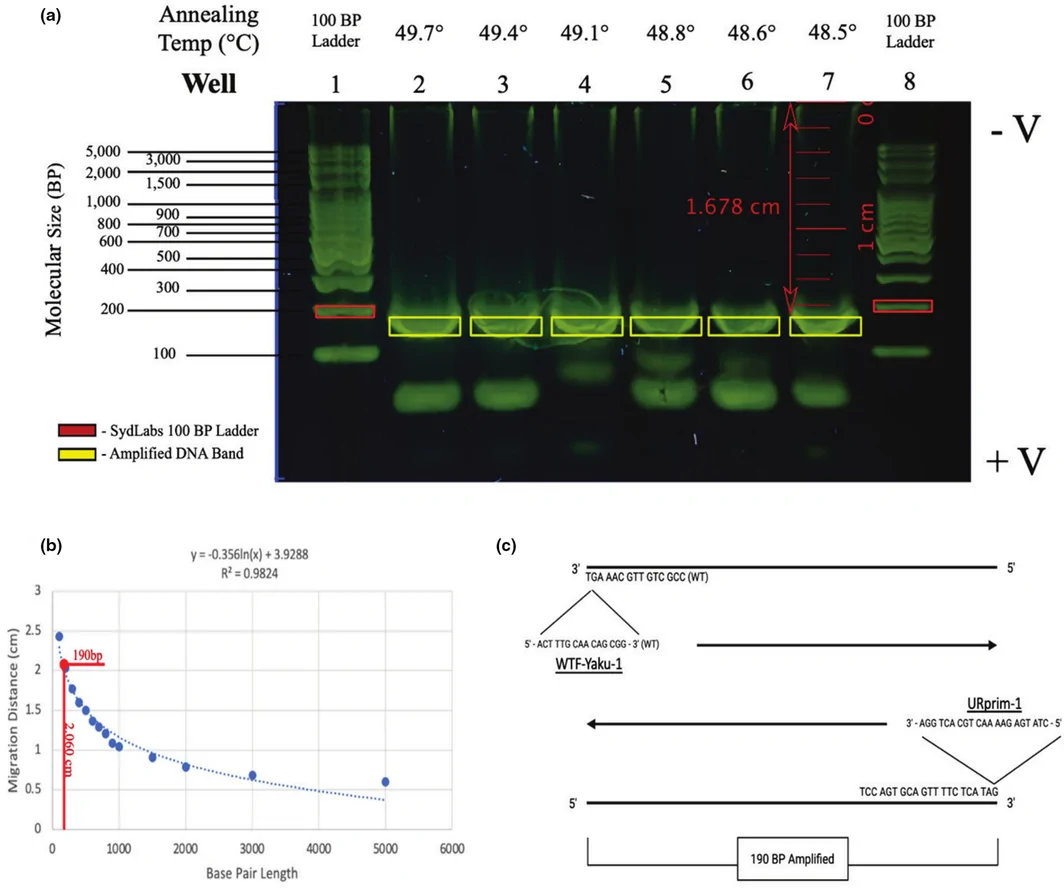
Experimental allele‐specific amplification and confirmation of wild‐type CFTR template using Yaku AS‐PCR. (a) Agarose gel showing amplification using wild‐type‐seeking primers. A single ~190 bp band appears only in reactions containing wild‐type CFTR DNA, while no amplification is observed with mutant template, supporting primer specificity. (b) Semi‐logarithmic regression analysis confirmed amplicon sizes of ~190 bp for the wild‐type–specific reactions, indicating that allele‐matched primers produced correctly sized products. (c) Mechanistic schematic of wild‐type primer‐template interactions at the W1282X locus, illustrating the binding pattern that supports exclusive amplification of the matching wild‐type template.

**FIGURE 5:**
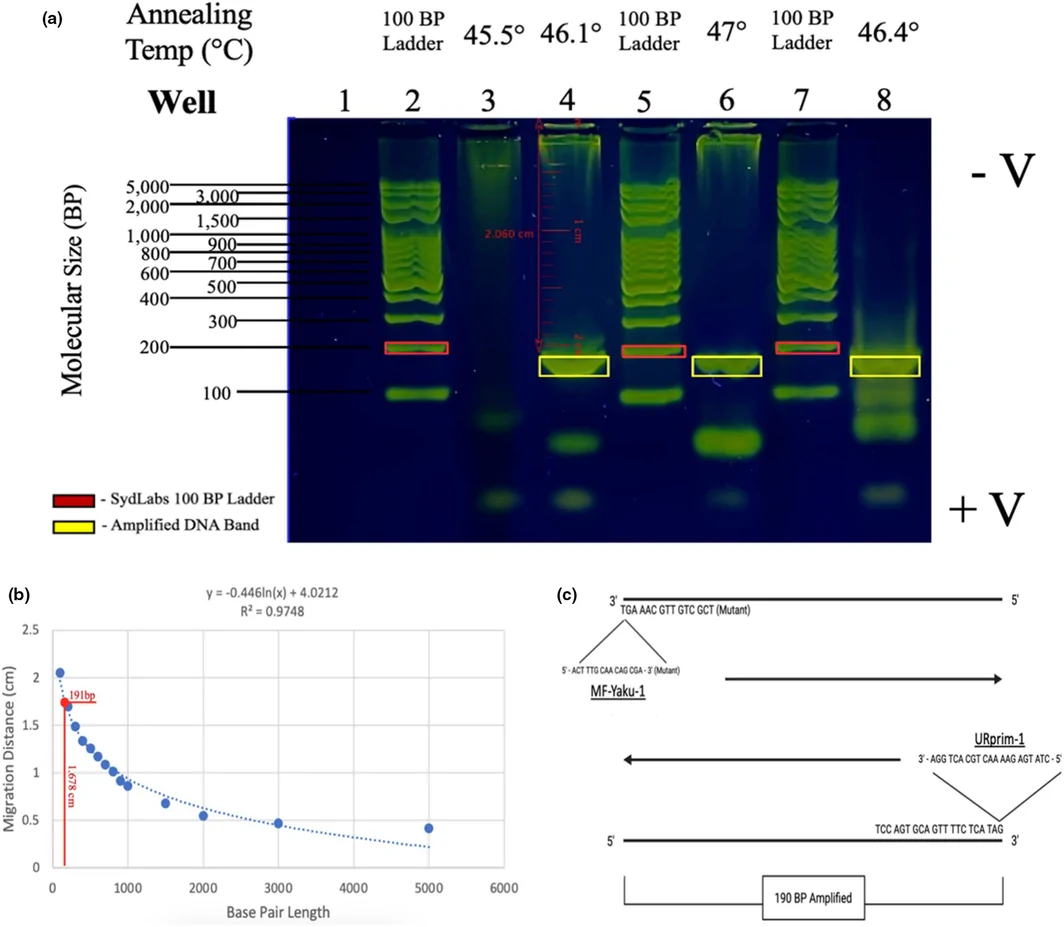
Experimental amplification and confirmation of W1282X mutant DNA using Yaku allele‐specific primers. (a) Agarose gel showing PCR amplicons generated from W1282X CFTR templates using Yaku‐designed primer sets. Distinct ~190 bp bands appear only when mutant‐seeking primers are paired with the complementary mutant template, confirming allele specificity and validating successful amplification across multiple experimental conditions. (b) Semi‐logarithmic plot comparing migration distances of observed amplicons to known ladder fragment lengths. The measured migration distance of 1.678 cm corresponded to a calculated product size of ~190 bp, supporting the expected amplicon length and reinforcing the reliability of the assay for distinguishing wild‐type and mutant DNA. (c) Mechanistic schematic of mutant primer‐template interactions at the W1282X locus, showing how correct terminal base pairing supports selective amplification of the mutant template.

### 3.5 Validation of methodology

A PCR forward primer targeting the G542X locus (ΔMF‐Yaku1) and a universal reverse primer (ΔPRprim1‐URP) were used in a PCR amplification to identify the G542X‐CFTR mutation in human genomic DNA as a validation. In gel lanes 3 and 4, with primer annealing temperatures of 59.3°C–62°C, an amplified band at ~300 bp was produced at the anticipated position (Figure 6a). A line of best fit equation was generated from the semi‐logarithmic analysis, where y represents migration distance and x represents the molecular size in base pairs. The migration distance of 1.905 cm yielded a calculated band size of 295 bp (Figure 6b).

**FIGURE 6:**
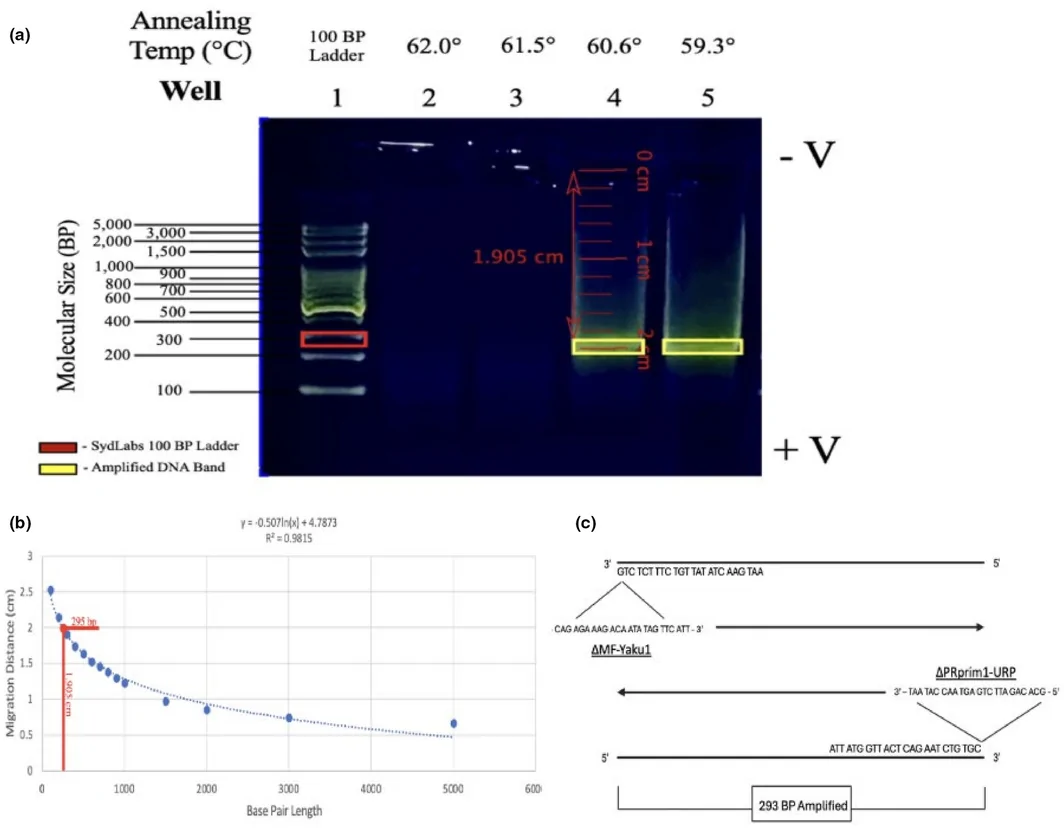
Further validation of the steric AS‐PCR methodology at an additional CFTR mutation site, G542X. (a) PCR products of G542X‐CFTR mutant human genomic DNA using the designed G542X primer (ΔMF‐Yaku1) and universal reverse (PRprim1‐URP) primers were targeted. Lane 1 contained a 100 bp ladder (SydLabs). Lanes 2–5 contained amplified PCR products with (ΔMF‐Yaku1, PRprim1‐URP) G542X mutant DNA. Lanes 3 and 4 produced a distinct band ~300 bp, confirming accurate primer annealing temperatures of 59.3°C–62°C and successful amplification of the mutant G542X‐CFTR gene fragment. This experiment prepared wells with 16 μL PCR cocktail and 4 μL bromophenol dye, with a 1.5% agarose gel run at 117 V for 30 min and visualized under UV illumination. (b) Semi‐logarithmic plot of migration distance versus fragment size using the 100 bp DNA ladder (SydLabs) as a reference. The amplified G542X‐CFTR band migrated 1.905 cm, corresponding to 295 bp as determined from the line‐of‐best‐fit equation y = −0.507ln(x) + 4.7873. This accurately represented the predicted product length. The strong agreement between predicted and observed fragment sizes confirmed primer accuracy and validated the use of Yaku steric designed primers for allele‐specific detection of the G452X mutation. (c) Mechanistic schematic of mutant primer‐template interactions at the G452X locus, showing how correct terminal base pairing supports selective amplification of the mutant template.

## 4 DISCUSSION

A recent “2026 Grand Challenges” initiative from the Gates Foundation sought to find Cost‐Disruptive Tools for Diagnosis, given that limited access to affordable diagnosis is a significant barrier to disease control in the world. This, of course, impacts both rural areas of the US, as well as low‐ and middle‐income countries. Thus, exploring and optimizing some traditional diagnostic approaches, which have become much more affordable, such as endpoint PCR, was explored in this study.

Polymerase Chain Reaction (PCR) is a method used for replication of a targeted DNA sequence using forward and reverse primers (Cruz‐Rangel et al., 2022). In recent decades, the costs associated with endpoint PCR have decreased significantly, making it a more accessible diagnostic. Yet in genetic diseases, a single‐base polymorphism (SNP) mutation is common and more difficult to diagnose with endpoint PCR. This study utilized more affordable endpoint PCR in a fashion to enhance DNA amplification sensitivity and SNP specificity by incorporating primary nested PCR products with sterically designed Yaku steric primers. Hidenobu Yaku's allele‐specific primer strategy (Yaku et al., 2008) was applied to design wild‐type and mutant forward primers that included a deliberate mismatch at the third position from the final 3′ terminal base. The primer's 3′ base is aligned with the SNP and confers base‐pair specificity. It was hypothesized that combining steric‐designed primers with nested PCR products would yield an accurate diagnostic assay for not just the W1282X CFTR mutation, but all point mutations. This study was intended as a proof‐of‐concept evaluation of allele‐specific endpoint PCR that could discriminate against the W128X SNP using conventional molecular laboratory equipment. By relying on a standard endpoint thermal cycler and agarose gel electrophoresis, the assay was developed with cost‐conscious implementation in mind and can provide a foundation for further optimization of reagent use and assay cost.

Cystic Fibrosis is an autosomal recessive genetic disorder frequently caused by SNP mutations in the cystic fibrosis transmembrane conductance regulator gene (Fanen et al., 2014). Impaired CFTR function disrupts ion transport and fluid homeostasis in epithelial tissues, resulting in thick mucus accumulation, chronic pulmonary infections, and progressive lung damage (Castellani & Assael, 2016). This disease affects approximately 80,000 individuals worldwide (Farinha & Callebaut, 2022). The W1282X SNP mutation is the sixth most common CFTR mutation worldwide, accounting for approximately 1.2% of mutant CFTR alleles (Michicich et al., 2025). The mutation arises from a G → A substitution at codon 1282 in exon 23, converting the tryptophan (W) codon TGG into the stop (X) codon TGA (Suzuki et al., 2022). This premature stop codon results in a truncated CFTR protein, impairing chloride ion transport across epithelial membranes and contributing to the clinical manifestations of cystic fibrosis.

### 4.1 Experimental design

This study aimed to aid the development of cost‐efficient allele‐specific primer strategies for detecting single‐nucleotide mutations using the more accessible conventional endpoint PCR in healthcare facilities worldwide. We hypothesized that allele‐specific primers designed using Yaku's intentional base pair mismatch steric strategy could selectively amplify DNA containing the W1282X mutation while preventing amplification of non‐matching wild‐type sequences. Detection reliability was enhanced through the use of a two‐strategy PCR approach. Primary PCR utilized designed nested forward and reverse primers (DnestF‐1 and DnestR‐1) to enhance the accuracy of the diagnostic test by creating a 591‐base template of the CFTR gene to be targeted in secondary endpoint PCR. Following initial amplification, secondary PCR employed designed wild‐type and mutant forward primers (WTF‐Yaku‐1 and MF‐Yaku‐1) alongside the universal reverse primer (URprim‐1) targeting the nested product. Allele‐specific primer design ensured that WTF‐Yaku‐1 annealed exclusively to the wild‐type DNA, whereas MF‐Yaku‐1 annealed exclusively to W1282X mutant template DNA. Prior research from the You et al. group was successful in detecting the W1282X mutation (You et al., 2024). This study was pursued to further test accessible strategies on the same SNP.

### 4.2 Ultimate findings

As a reagent control, the Rz gene of the lambda viral bacteriophage genome was amplified. A ~395 bp PCR product supported that the PCR reagent functional status was nominal.

As a template control, the CFTR gene of human purified genome was amplified with PCR primers (Fprim‐1 and URprim‐1) published by Feriotto et al. (2001). Successful amplification of a ~472 bp PCR product supported genome integrity as intact.

A PCR approach was designed to successfully integrate a nested primer step with allele‐specific Yaku‐designed primers to support the detection of the W1282X mutation with endpoint PCR. Following generation of the 591 base pair nested PCR product, secondary PCR amplification with the designed primers produced allele‐specific bands that were visualized by gel electrophoresis. Analysis of the gel under the UV transilluminator revealed bands around 190 base pairs, which were further confirmed by semi‐logarithmic analysis. The results were supportive of W1282X‐CFTR SNP allele‐specific detection using the combined two‐strategy PCR approach in endpoint thermal cyclers. This approach was then successfully validated via replication with another SNP mutation, the G542X‐CFTR SNP.

### 4.3 Future directions

This assay can be expanded to distinguish wild‐type and mutant alleles associated with other genetic diseases, particularly those caused by point mutations. In addition, this builds upon prior work by the You group, which also successfully targeted the W1282X locus with endpoint PCR. The present study used a different primer configuration and a shorter expected amplicon size, further supporting the idea that endpoint assay designs may be effective for affordable diagnosis. Because this approach relies on affordable PCR and gel electrophoresis rather than more advanced instrumentation, it may provide a more accessible and cost‐effective option for SNP screening in many clinical‐based laboratories with limited resources in the US and elsewhere. Overall, these findings support the continued development of allele‐specific PCR strategies for practical genetic mutation detection for human, bacterial, and viral DNA sequence targets in diagnosis.

## AUTHOR CONTRIBUTIONS

**John A. Torok IV:** Conceptualization; data curation; formal analysis; funding acquisition; investigation; methodology; project administration; resources; supervision. **Derrick L. Brown:** Conceptualization; data curation; formal analysis; funding acquisition; investigation; methodology; project administration; resources; supervision. **Michael R. Grassl:** Conceptualization; data curation; formal analysis; funding acquisition; investigation; methodology; project administration; resources; supervision. **Samyuktha Vijayakumar:** Conceptualization; data curation; formal analysis; funding acquisition; investigation; methodology; project administration; resources; supervision. **Kierin P. Patel:** Conceptualization; data curation; formal analysis; funding acquisition; investigation; methodology; project administration; resources; supervision. **Ayaan Jehangir:** Conceptualization; data curation; formal analysis; funding acquisition; investigation; methodology; project administration; resources; supervision. **Jay Katti:** Conceptualization; data curation; formal analysis; funding acquisition; investigation; methodology; project administration; resources; supervision. **Daniel F. Barnas II:** Conceptualization; data curation; formal analysis; funding acquisition; investigation; methodology; project administration; resources; supervision. **Logan P. Dooley:** Conceptualization; data curation; formal analysis; funding acquisition; investigation; methodology; project administration; resources; supervision. **Anahita Dhar:** Conceptualization; data curation; formal analysis; funding acquisition; investigation; methodology; project administration; resources; supervision. **Chase A. MacDougall:** Conceptualization; data curation; formal analysis; funding acquisition; investigation; methodology; project administration; resources; supervision. **Ryan J. Ciacci:** Conceptualization; data curation; formal analysis; funding acquisition; investigation; methodology; project administration; resources; supervision. **Siersha R. Torres:** Conceptualization; data curation; formal analysis; funding acquisition; investigation; methodology; project administration; resources; supervision. **Nicole Katrib:** Conceptualization; data curation; formal analysis; funding acquisition; investigation; methodology; project administration; resources; supervision. **Douglas Luckie:** Conceptualization; data curation; formal analysis; funding acquisition; investigation; methodology; project administration; resources; supervision.

## FUNDING INFORMATION

Our CF and STEM learning laboratories are supported by Cystic Fibrosis Foundation and Pennsylvania Cystic Fibrosis Inc. grants (to D.L.) for our physiology research program and by grants from the National Science Foundation (to D.L.) for our education research program.

## CONFLICT OF INTEREST STATEMENT

No conflicts of interest, financial or otherwise, are declared by the authors.

## ETHICS STATEMENT

No human or animal participants were involved in this study.

## ARTIFICIAL INTELLIGENCE GENERATED CONTENT (AIGC)

No generative artificial intelligence tools were used in the preparation of this manuscript.

## Data Availability

Data analyzed during this study is included in this published article.
